# Interaction of Antibiotics and Humic Substances: Environmental Consequences and Remediation Prospects

**DOI:** 10.3390/molecules27227754

**Published:** 2022-11-10

**Authors:** Natalia A. Kulikova, Alexandra A. Solovyova, Irina V. Perminova

**Affiliations:** 1 Department of Soil Science, Lomonosov Moscow State University, Leninskiye Gory 1-12, 119991 Moscow, Russia; 2 Bach Institute of Biochemistry, Fundamentals of Biotechnology Federal Research Center, Russian Academy of Sciences, pr. Leninskiy 33, 119071 Moscow, Russia; 3 Department of Chemistry, Lomonosov Moscow State University, Leninskiye Gory 1-3, 119991 Moscow, Russia

**Keywords:** pollution, sorption, binding constant, mobile genetic elements

## Abstract

The occurrence and distribution of antibiotics in the environment has received increasing attention due to their potential adverse effects on human health and ecosystems. Humic substances (HS) influence the mobility, reactivity, and bioavailability of antibiotics in the environment significantly due to their interaction. As a result, HS can affect the dissemination of antibiotic-resistance genes, which is one of the main problems arising from contamination with antibiotics. The review provides quantitative data on the binding of HS with fluoroquinolones, macrolides, sulfonamides, and tetracyclines and reports the proposed mechanisms of their interaction. The main issues of the quantification of antibiotic–HS interaction are discussed, which are a development of standard approaches and the accumulation of a dataset using a standard methodology. This would allow the implementation of a meta-analysis of data to reveal the patterns of the binding of antibiotics to HS. Examples of successful development of humic-based sorbents for fluoroquinolone and tetracycline removal from environmental water systems or polluted wastewaters were given. Data on the various effects of HS on the dissemination of antibiotic-resistance genes (ARGs) were summarized. The detailed characterization of HS properties as a key point of assessing the environmental consequences of the formation of antibiotic–HS complexes, such as the dissemination of antibiotic resistance, was proposed.

## 1. Introduction

Antibiotics are a substance able to inhibit or kill microorganisms [1]. As emerging contaminants, the occurrence and distribution of antibiotics in the environment has received increasing attention due to their potential adverse effects on human health and the ecosystem [2]. Modern antibiotics are usually classified by their chemical structure. The top 10 antibiotic classes include penicillins (β-lactams), tetracyclines, cephalosporins, quinolones (fluoroquinolones), lincomycins, macrolides, sulfonamides, glycopeptides, aminoglycosides, and carbapenems [3]. The remnants of antibiotics are released into the environment through effluents and human wastes inducing aquatic and soil contamination [4]. According to existing estimates, about 20–97% of any doses of most antibiotics administered to humans and animals is excreted as an unmetabolized active substance, eventually reaching urban and hospital wastewaters [5]. As a result, antibiotics have been frequently detected in the effluents of municipal wastewater treatment plants, secondary sludge and biosolids, surface water, groundwater, drinking water, and soil and sediments [6].

Global antibiotic consumption by humans alone increased by 36% between 2000 and 2010 [7] and worldwide, antibiotic usage lies between 100,000 and 200,000 tons per year [8]. The current coronavirus disease 2019 (COVID-19) has led to an even greater increase in antibiotic use: a recent rapid review and meta-analysis including 154 studies with available data from 30,623 patients showed that the prevalence of antibiotic prescription was 74.6% [9]. An increase in the antimicrobial consumption during the first wave of the COVID-19 pandemic was noticed for ceftriaxone, carbapenems, daptomycin, azithromycin, and linezolid [10]. These facts illustrate that antibiotic pollution is an ever-growing problem.

As in the case of many other organic pollutants, the behavior of antibiotics is significantly influenced by natural organic matter and, in particular, its most reactive component—humic substances (HS). The mobility, reactivity, and bioavailability of antibiotics in the environment are influenced by HS [11,12,13]. Numerous studies have shown that HS can sorb antibiotics [14,15,16,17,18,19,20,21,22,23,24,25,26,27,28,29,30] and affect their decomposition [31,32,33,34,35,36,37,38,39,40,41,42,43,44,45,46,47] and toxicity [15,48]. The resulting effect of HS is a decrease in the relative content of antibiotic-resistance genes (ARGs) [49,50,51], the increase of which in natural environments is the main problem arising from contamination with antibiotics [4,52]. Therefore, the study of the interaction of HS has become increasingly relevant in terms of the development of technologies for wastewater treatment from antibiotics [26,29,49,53,54,55,56,57,58,59,60,61]. To date, a number of sorbents have already been created for which the high efficiency of removing antibiotics has been demonstrated [29,61,62,63,64,65].

With this in view, the review paper focused on the systematization and analysis of data on (1) the effect of HS on the main processes of transformation of antibiotics in nature and (2) the technologies developed on their basis for the purification of media contaminated with antibiotics.

## 2. The Sources and Fate of Antibiotic Pollution in the Environment

Three major pathways for antibiotics entering into the environment are generally considered: effluents from Wastewater Treatment Plants (WWTPs), chemical manufacturing plants, and animal husbandry and aquaculture [66]. However, the list of potential sources of antibiotics is much broader and also includes households, hospitals, agricultural and raw wastewater sewer leakage, surface runoff, and other discharges [4,6,67,68]. The emerging antibiotics continue to persist in the ecosystem due to their lower degradation rates, improper disposal, misuse, bulk manufacturing, and wastage [69]. The occurrence and distribution of pharmaceutical compounds in different environments is examined in detail in many studies [6,52,69,70,71,72,73].

A tremendous amount of attention to the issue of environmental pollution with antibiotics in recent years is due to the negative consequences of their presence in nature. Nowadays, antibiotics are being considered as ubiquitously occurring persistent bioactive chemicals that are potentially hazardous to soil bacteria and other organisms [74]. The main issues related to the presence of antibiotics in the environment are as follows:

- development of antibiotic resistance [4,52,68,70];
- disruption microbial communities by favoring the growth of resistant or tolerant microbial lineages [68];
- negative impact on freshwater organisms including bacteria, cyanobacteria, diatom algae, plants, crustacean, mollusks, and fish [66,67,75];
- negative impacts on soil microorganisms and soil enzyme activity [72,74,76];
- phytotoxicity in relation to crops [74,77];
- vegetable pollution with antibiotics [74,78,79].

The most important issue of antibiotic release into the environment is related to the development of antibiotic resistance, which has resulted in the reduction of therapeutic potential against human and animal pathogens [4,52,70]. Antibiotics can accumulate in food webs and, even more alarmingly, ARGs can be transferred between environmental bacteria and human pathogens [52]. Recently, ARGs were detected against seven commonly used antibiotics in the particulate matter from city air worldwide [80]. According to recent reports, 23,000 people in the USA may have died each year due to antibiotic-resistant infections, while in the European Union (EU) this problem may be the cause of 25,000 deaths per year. Globally, a conservative estimate suggests that 700,000 people die each year due to antibiotic resistance [67].

Among antibiotic classes, fluoroquinolones (FQ), macrolides (MA), sulfonamides (SU), and tetracyclines (TE) can be detected in waste effluents most frequently [4]. Their concentrations in surface and wastewaters vary in the range of μg/L to ng/L and their presence is addressed in all continents (Table 1).

The data presented in Table 1 show that despite the recognized need for the monitoring of environmental pollution with antibiotics, such monitoring is not carried out in many countries because antibiotic pollution is poorly regulated on a local and global scale [82]. This is also indicated by an extremely limited amount of data on the content of synthetic antibiotics in soils. In the detailed overview of ref. [6], data for only seven countries were presented (China, France, Germany, Iran, Malaysia, South Korea, and USA). According to the data provided, the content of FQ, MA, SU, and TE in manure and manure-amended soils varies in the range 0.053–225.6, 0.007–0.16, 0.00029–46.4, and 0.00013–3746.4 mg/kg dry weight, respectively. The limited data available are also indicated by the fact that in some cases the concentrations of antibiotics in surface waters exceed those in wastewater (Table 1). On the other hand, the latter may relate to wastewater treatment problems when removing antibiotics, as they require special degradation treatments for effluents and permit their reuse in various aspects [4,67,83].

The life-cycle of antibiotics in the environment is governed by a number of biological and physicochemical processes in soil–water systems, and these compounds may persist through a cycle of partial transformation, bioaccumulation, and gradual deposition in soil, surface water, and groundwater [67]. Elimination of antibiotics entering into the environment occurs via several processes, and sorption, photodegradation, biodegradation, and oxidation appear to be the most significant [70]. In the aquatic environments, antibiotics become mixed and are transported downstream. During mixing and transportation, antibiotics may become degraded, adsorbed to suspended matter, may accumulate onto sediments, and may return to the water column by resuspension [70]. In the soil environment, the persistence of an antibiotic mostly depends on its photostability, binding/adsorption capability, degradation rate, and leaching in water [70]. The behavior of a particular antibiotic in the environment is determined by its physical and chemical properties.

## 3. Properties of Antibiotics

**Figure 1:**
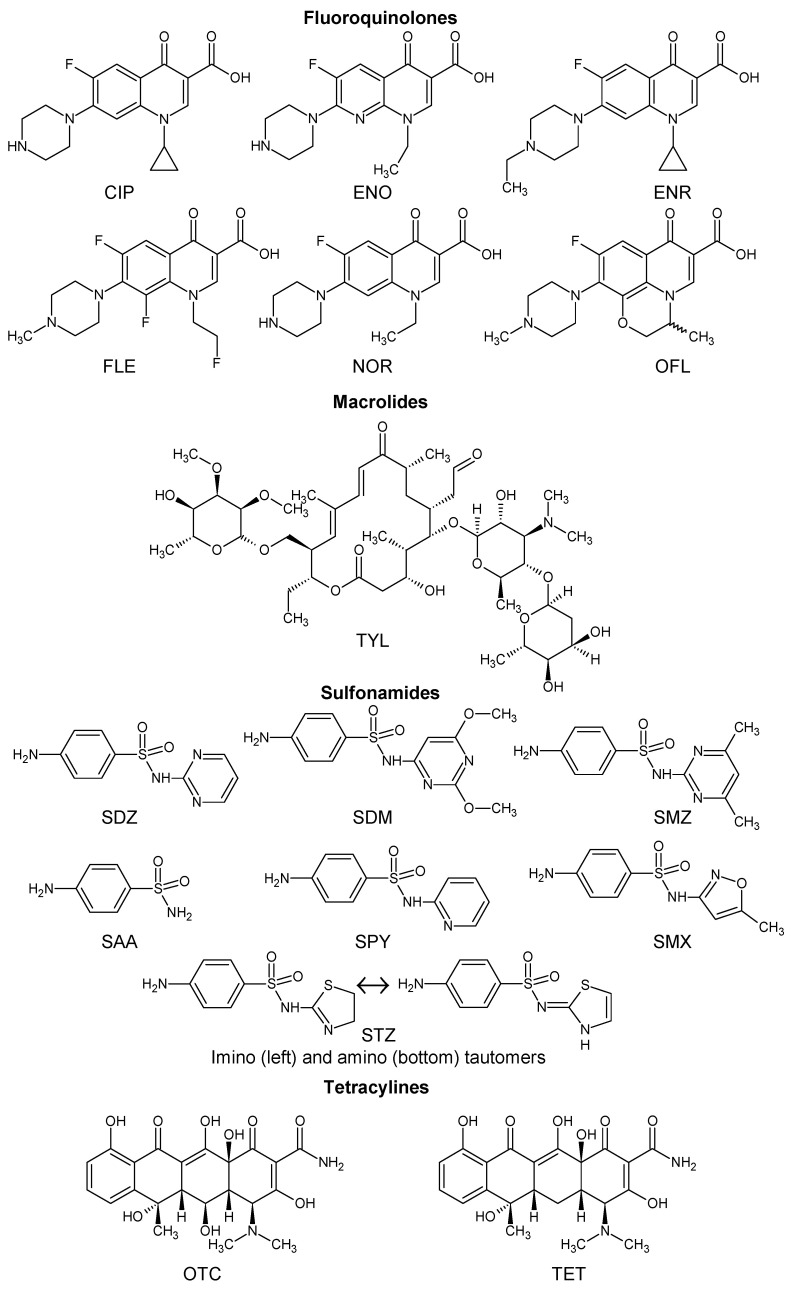
Skeletal formulas of the selected antibiotics considered in this review. FQ: ciprofloxacin (CIP); enoxacin (ENO); enrofloxacin (ENR); fleroxacin (FLE); norfloxacin (NOR); ofloxacin (OFL). MA: tylosin (TYL). SU: sulfadiazine (SDZ); sulfadimethoxine (SDM); sulfamethazine (SMZ); sulfamethoxazole (SMX); sulfanilamide (SAA); sulfapyridine (SPY); sulfathiazole (STZ). TE: oxytetracycline (OTC); tetracycline (TET).

Currently, the European Committee on Antimicrobial Susceptibility Testing (EUCAST) database contains information on more than 100 different antibiotics [84], but only a few of them have been systematically studied in terms of their interaction with HS. Despite the relatively small number of antibiotics studied, it should be noted that their list coincides well with the list of antibiotics presented in Table 1. This indicates the relevance of the research conducted in this area. Structures of the selected target antibiotics belonging to different classes considered in this review are provided in Figure 1.

Table 2 denotes the list and physiochemical properties of the antibiotics mentioned in this review.

**Figure 2:**
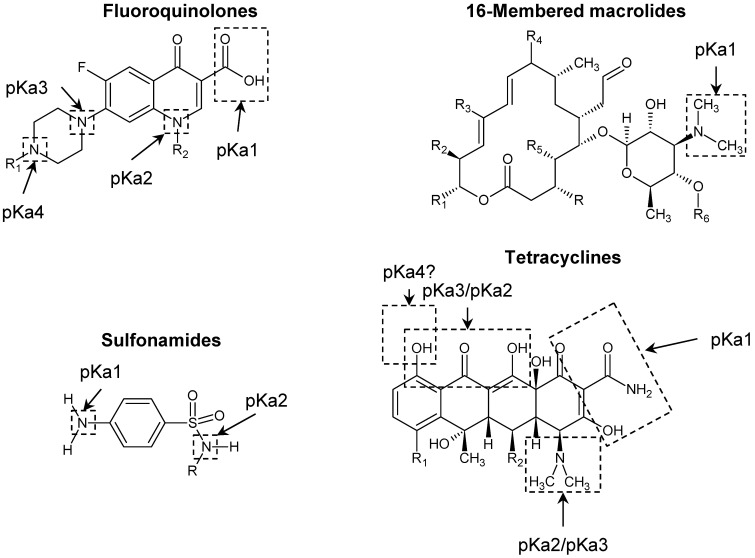
Specific moieties corresponding to the pKa values of the generic structures of the selected classes of antibiotics considered in this review.

Figure 1 demonstrates that antibiotics are ionic organic pollutants containing one or more functional groups, such as carboxyl or amino, belonging to the hydrophilic substances [27]. The generic structure of the fluoroquinolone antibiotics (Figure 2) highlights the requirement for at least one fluorine in the aromatic ring. Introducing fluorine into a benzene ring is well known to have a positive impact on the molecule’s lipophilicity. It is believed that fluorine facilitates an antibiotic’s binding and cellular penetration [93]. According to the values of logKow presented in Table 2, the selected fluoroquinolones are highly soluble substances mainly of low hydrophobicity. These compounds contain a carboxylic group and three basic nitrogen sites, thus possibly exhibiting four pKa values at maximum [86].

The pKa1 of fluoroquinolones is assigned to the carboxylic group attached to the benzene ring, while it is difficult to assign the remaining three pKa values to specific nitrogen sites [94]. However, based on the electron density of the three nitrogen sites, Qiang and Adams suggested a specific distribution of constants in groups (Figure 2) [86]. Taking into account the values of pKa presented in the Table 2, it can be assumed that under the environmentally relevant pH conditions, fluoroquinolone antibiotics are mainly in the form of a two-charge cation FQ^2+^ (pH 4–5) or a single-charge cation FQ^+^ (pH 7–8). At pH values close to neutral, these antibiotics do not seem to carry a charge, as indicated by the values of their isoelectric point, which is in the range from 6.9 to 7.4 (Table 2). Moreover, the presence of carboxylic and piperazinyl groups allow the molecules of fluoroquinolones to exist in solution in zwitterionic form [95].

A 16-membered macrolide, TYL, is tylonolide, with mono- and diglycosyl moieties attached to two of its hydroxy groups (Figure 2). It is a weakly alkaline compound containing a basic dimethylamine (−N(CH_3_)_2_) group which is able to gain a proton and so this particular group is believed to correspond to the pKa of TYL [86] (Figure 2). Under the pH range 6–8, TYL seemingly occurs roughly equally in the TYL^+^ and TYL^0^ species, while at pH < 6 it can be found mainly as TYL^+^ [19]. Compared with other antibiotics, tylosin has a high hydrophobicity (Table 2).

A sulfonamide antibiotic molecule contains one basic amine group (−NH_2_) that is able to gain a proton and one acidic amide group (–NH–), which correspond to pKa1 and pKa2, respectively [86] (Figure 2). At pH values above 7–8, these compounds exist primarily as anions SU^−^, while the cationic form of sulfonamides SU^+^ can be detected at very low pH values of less than 3 [19]. Some sulfonamides have been reported to be amphoteric. For example, at pH values close to pI, SMZ acts as a zwitterion due to the protonation of −NH_2_ and the deprotonation of −NH [96].

Tetracyclines are hydrophilic antibiotics possessing three dissociation constants of approximately 3, 8, and 9. The first acid dissociation constant of tetracyclines is attributed to the hydroxy group of the tricarbonyl system [92]. Some researchers have attempted to assign the other pKa values to particular functional moieties in the tetracycline molecule, but this often led to conflicting results for pKa2 and pKa3 [86]. Investigation of the microscopic ionization constants for tetracyclines through fluorescence measurements allowed Bhatt and Jee [97] to propose assigning pKa2 and pKa3, as depicted in Figure 2. Some researchers also reported on pKa4 values in the range 10.7–12.0, which is associated with the phenolic hydroxy group in the benzene ring [92]. Therefore TE possesses an amphoteric character, being in the anionic form at alkaline pH values and in the cationic form at acidic pH values. At a pH value near the isoelectric point, 4–6, TE exists in the zwitterionic form [18,92].

Therefore, except for FQ, the antibiotics presented in Table 2 are mostly bear neutral or negatively charged under the environmentally relevant pH values, while single- or double-charged cations can be found under these conditions for FQ. Apart from TYL, many of them possess both basic and acidic groups and can exist in the form of zwitterions in the pH range 4–8. Besides, FQ, SU, and TE have a benzene ring in their structure, enabling Pi-stacking.

## 4. Sorption of Antibiotics by HS

### 4.1. Quantitative Characteristics of Antibiotic Sorption by HS

There is currently no unified method for the study of binding of antibiotics to HS. To quantify the strength of binding process, the Stern–Volmer constant K_SV_ or binding constant K_b_, distribution coefficient K_d_, Freundlich constant K_F_, Langmuir constant K_L_, and maximum adsorption b can be used. Both HS in the dissolved and solid forms can be used for experiments, and the fluorescence-quenching technique and batch equilibrium experiments are two main approaches.

Fluorescence quenching is a useful technique in studying the interaction between a fluorophore-containing antibiotic (AB) and its quencher (HS). Quenching refers to various processes that decrease the fluorescence intensity, including excited state reactions, energy transfer, and static and dynamic quenching. The main quenching mechanism for antibiotic–HS interaction is supposed to be static quenching [27], occurring when AB and HS bind to create a less fluorescent complex AB–HS. In the formation of this complex, the binding equilibrium Stern–Volmer constant K_SV_ can be determined through linear regression as follows [17]:

(1) $$\frac{F_{0}}{F}=1+K_{\mathrm{SV}}\times \left[\mathrm{HS}\right]$$

where F_0_ is the fluorescence intensity of the antibiotic alone, F is the fluorescence intensity of fluorophore with HS added, and [HS] is the equilibrium concentration of the HS. Due to HS possessing intrinsic fluorescence at the excitation wavelength of antibiotics, the fluorescence values obtained from HS solutions from the fluorescence values obtained for antibiotic quenching to remove background effects can be also found [98].

In addition, in the static quenching process, the Stern–Volmer equation changes as the site-binding equation [99]:

(2) $$\log\left(\frac{F_{0}-F}{F}\right)={\mathrm{logK}}_{b}+x\times \log\left[\mathrm{HS}\right]$$

where K_b_ is the binding constant and x is the binding site number. If the appropriate HS concentration range that provides a linear Stern–Volmer plot is selected, the binding constant and sorption site amounts can be evaluated by comparing the intercept and slope of the plot directly [27]. Though the fluorescence-quenching method has become one of the most popular techniques because of its simplicity and elegance, it is limited to fluorescent compounds and has been shown to overestimate binding coefficient values [100].

In batch equilibrium experiments, HS solids are mixed with antibiotic solution followed by the determination of the residual aqueous-phase solute concentration after a certain period [22,30]. To remove HS particles, filtration through a 0.22 μm membrane can be applied [22,30]. If HS are used as a solute, to separate free and bound HS–antibiotics, dialysis systems [18,23] or solid-phase extraction [16] can be used. The concentration on non-bound antibiotics can be further estimated using chromatographic methods [22,30] or the fluorescence-quenching approach [21,27].

Plotting the equilibrium amount of the sorbed antibiotic AB_S_ against its equilibrium concentration [AB] yields the sorption isotherm. The sorption isotherm is fitted to the Langmuir or Freundlich equation. The Langmuir isotherm equation is expressed as

(3) $${\mathrm{AB}}_{s}=\frac{K_{L}\times \left[\mathrm{AB}\right]\times b}{1+K_{L}\times \left[\mathrm{AB}\right]}$$

where AB_S_ is the equilibrium solid phase content of the antibiotic, [AB] is the equilibrium aqueous phase concentrations, and K_L_ and b are the Langmuir constants and maximum sorption, respectively.

The Freundlich isotherm equation is expressed as

(4) $${\mathrm{AB}}_{s}=K_{F}\times {\left[\mathrm{AB}\right]}^{n}$$

where K_F_ and n are the Freundlich equilibrium sorption constants and nonlinearity, respectively.

When the sorption isotherm is linear or when a single-point estimation is performed, the strength of an antibiotic sorption onto HS can be represented by sorption distribution coefficient K_d_:

(5) $$K_{d}=\frac{{\mathrm{AB}}_{s}}{\left[\mathrm{AB}\right]}$$

The unit of measurement of the obtained constants depends on which units of measurement have been used to express the concentrations of HS and antibiotics.

Recently, fitting adsorption isotherms to the Polanyi–Manes model was also proposed [26]:

(6) $$\log\left({\mathrm{AB}}_{S}\right)=\log\left(b\right)+Z\times {\left(R\times T\times \ln\left(\frac{S}{\left[\mathrm{AB}\right]}\right)\right)}^{a}$$

where R is the universal gas constant, T is the absolution temperature, S is the maximum solubility of the antibiotic, and Z and a are fitting parameters of the Polanyi–Manes model, respectively.

The published constants describing the strength of binding of antibiotics to HS are given in Table 3.

In general, the analysis of the data in Table 3 allows us to conclude that fluoroquinolone antibiotics interact most intensively with HS. The binding constant K_b_ demonstrates the higher affinity of HS to fluoroquinolones as compared with sulfonamides. Similar findings can be found from the data on distribution coefficient K_d_ for fluoroquinolone NOR, though CIP showed values of K_d_ close to that for SMZ, SMX, and STZ.

The Freundlich constants cannot be used directly to compare binding affinity due to their dimension variation depending nonlinearity n. However, the application of this isotherm allows characterizing the nonlinearity of the antibiotics’ binding to HS. The closeness of the value n to 1, which indicates an almost linear isotherm, was observed only for a limited number of antibiotics. They were CIP [14] and TET [18,25]. The nonlinearity apparently demonstrates that linear partitioning or site complexation cannot fully describe the interactions of antibiotics with humic material, and site-specific interactions at limited interior or external molecular surfaces should be also considered [100].

Table 3 demonstrates a wide variety of approaches and conditions used to assess the binding strength of antibiotics to HS, though it is difficult to draw any unambiguous conclusions based on the data presented in Table 3. To generalize the existing data, we recalculated the constants given in the literature to uniform dimensions and determined their ranges (Table 4).

Data from Table 4 indicate an intense interaction of antibiotics with HS, sometimes comparable to polynuclear aromatic hydrocarbons (PAH) and hydrophobic organic contaminants (HOC). The study of partitioning of HOC 4-monochlorobiphenyl (MCB) to dissolved organic matter demonstrated values of the distribution coefficient K_d_ for this compound as high as 8318 L kg^−1^ [102]. The reported range for K_d_ for PAH pyrene to lake aquatic humic matter were 2930–44,490 L kg^−1^ [103]. Similar values of K_d_ could be found for fluoroquinolones NOR, OFL, and TET (Table 4). The reported distribution constants for macrolide TYL and sulfonamide SMX and SMZ were generally an order of magnitude lower (Table 4).

In general, Table 4 clearly demonstrates that data on the interaction of antibiotics with HS are still limited. The interaction of fluoroquinolone antibiotics with HS has been investigated quite often, but macrolide and sulfonamide are much less studied. Besides, usage of various types of the isotherms to quantify antibiotic–HS binding results in the impossibility of comparing the obtained constants. For example, the interaction of HS with sulfanilamides is mainly quantified in terms of the Freundlich isotherm, while with fluoroquinolones are by binding or Stern–Volmer constants. Therefore, additional experiments are required.

Another important issue is the extremely wide range of the reported constants, which seemingly resulted from the different experimental conditions used.

### 4.2. Discussion of the Quantification of Antibiotics—HS Interaction: Main Issues

When studying the interactions of HS with antibiotics, the used methods and approaches vary greatly. This often makes it difficult to compare the results. For example, the initial concentrations of FQ, MA, SU, and TE are in the range 0.05–940, 0.5–50, 0.0005–372, and 0.04–44 mg/L, respectively (Table 4). However, these parameters drastically alter the distribution coefficient when single-point estimation is performed. Wang and co-authors reported the K_d_ values for OFL binding by HA at the equilibrium concentrations of antibiotic of 1 and 10 mg L^−1^, and they were 5570 and 14,300 L kg^−1^, respectively [26]. The ratio of mass concentrations of HS to the antibiotic varies from 0.01 [21,30] to more than 12 million [23,30], and HS can be presented in both dissolved and solid form. This variety of approaches indicates the need to develop standard terms and conditions for obtaining quantitative estimates of the interaction of HS with antibiotics.

Another important issue of standardization is the choice of the method of the physical separation of the adsorptive from the adsorbent. One reliable approach for elucidating values for binding constants of organic substances to HS without the need for physical separation is the fluorescence-quenching technique due to its sensitivity, nondestructivity, fast and easy operation, and requirement of only small quantities of a sample [101,103]. However, fluorescence-quenching-derived constant values are, as a rule, significantly larger than those measured using other methods. This fact was demonstrated for PAH [103]. In addition, the use of fluorescence quenching is only possible if the intensity of the fluorescence of the antibiotic exceeds the intensity of the fluorescence quenching of HS by several times. Therefore, in some cases, it is necessary to separate the bound and free antibiotics. Filtration [22], centrifugation [19,20,25,26,28], extraction [24], equilibrium dialysis [18,23], and solid-phase extraction [15,16] are used to separate bound and free antibiotics. No systematic study has aimed to compare binding constants derived using different approaches. However, some reported examinations of various techniques used to determine the association of antibiotics with HS revealed a crucial role of the method. Ding et al. determined the binding of TET with dissolved HS using solid-phase extraction and validated the results by comparison with the results measured using the common equilibrium dialysis technique [16]. For the solid-phase extraction method, about 7.8 mg of TET was bound per 1 g of HS, while the equilibrium dialysis technique manifested about 6.2 mg g^−1^. The authors concluded the difference of the antibiotic affinity with HS for the equilibrium dialysis method, due to the Donnan effect, resulted in the uneven distributions of freely membrane-penetrating TET and protons inside vs. outside of the dialysis cell.

Thus, there are two main areas that need to be developed: the development of standard approaches and the accumulation of a dataset using a standard methodology. It should be noted, however, that the use of various conditions in determining the binding of antibiotics to HS provides the understanding of the mechanisms of this interaction.

### 4.3. Putative Mechanisms of HS and NOM Interaction with Antibiotics

As can be seen from the data presented in Table 3, most studies have been conducted with variable temperature, pH, or ionic strength. Additionally, metals were added in the reaction media in some cases. Based on the changes in the constants of the interaction of antibiotics with HS under varied conditions, assumptions were made about the possible mechanisms of the interaction (Table 5).

Additionally, a study of antibiotic–HS complexes using nuclear magnetic resonance (NMR), ultra violet-visible (UV-vis), and Fourier transform infrared (FTIR) techniques can be applied to prove the proposed mechanisms [28,101,108].

#### 4.3.1. Temperature Effect

The monitoring of the binding of antibiotics with HS at varied temperatures when using the fluorescence-quenching approach provides information regarding whether static or dynamic quenching is the main quenching process in the system. In the dynamic quenching process, a temperature increase leads to an increased collisional frequency and thus the K_SV_. In the static quenching process, on the other hand, the temperature increase tends to dissociate the fluorophore–quencher complex, resulting in a decrease of K_SV_ [27]. In the vast majority of studies, the K_SV_ values of antibiotic–HS systems decrease with increasing temperature. This has been demonstrated for fluoroquinolones (CIP [22,101], ENO [17], NOR [17,28]), tetracyclines (OTC [27]), and some sulfonamides (SDZ [27]). Therefore, static quenching due to antibiotic–HS complex formation was proposed in this case. For macrolides (TYL [19]) and some sulfonamides (SMZ [19] and SMX [22]), the sorption increased with increasing temperature, which indicated that higher temperatures could favor the sorption of these antibiotics by HS.

To further consider the nature of intermolecular forces, the thermodynamic parameters can be determined from the temperature dependence, including the enthalpy changes ΔH, the entropy change ΔS, and the free-energy change ΔG.

Gibbs free-energy change can be calculated using the Gibbs free-energy isotherm equation:

(7) ΔG=−R×T×lnK

where R is the ideal gas constant, T is the absolute temperature in Kelvin, and K is the distribution coefficient K_d_ or the binding constant K_b_ [27]. The negative values of ΔG indicate that the sorption process is thermodynamically favorable and spontaneous [28].

On the other hand, the Gibbs free energy of reaction can be expressed in terms of the enthalpy change ΔH and the entropy change ΔS using the deformed van’t Hoff equation [27]:

(8) ΔG=ΔH−T×ΔS

Combining (7) and (8), one can obtain

(9) $$\mathrm{lnK}=-\frac{\Delta H}{R\times T}+\frac{\Delta S}{R}$$

Provided that ΔH and ΔS are constant, the preceding equation provides lnK as a linear function of 1/T, and the latter is known as the linear form of the van’t Hoff equation. The slope of the line may be multiplied by the ideal gas constant R to obtain ΔH, and the intercept may be multiplied by R to obtain ΔS.

In accordance with the attribution of thermodynamic parameters to various types of interactions summarized by Ross and Subramanian [109], positive values of ΔH and ΔS relate to hydrophobic interaction as the main force; negative values of ΔH and ΔS indicate van der Waals and H-bond formation, respectively. If ΔS is positive and ΔH is negative or slightly positive, ionic interaction is supposed to play a key role. In addition, changes in ΔH may allow distinguishing the physical sorption (ΔH < 40 kJ mol^−1^) and chemisorption (ΔH > 40 kJ mol^−1^) [19,28].

The fluoroquinolones CIP, ENO, ENR, FLE, NOR, and OFL were demonstrated to have the negative values ΔH and ΔS for the interactions with HS, indicating both van der Waals force and hydrogen bonding were responsible for the reactions [17,101]. However, Zhang and co-authors reported the negative value of ΔH and the slight positive value of ΔS for the process of sorption of NOR by coal HS [28]. Therefore, the ionic interaction between NOR and HS can be hypothesized.

The negative values of ΔH for the fluoroquinolones and HS binding were also attributed to the electrostatic interaction [101]. The negative values ΔH and ΔS were also reported for tetracycline OTC and sulfonamide SDZ [27]. For the interaction between HS and macrolide TYL and sulfonamide SMZ, Guo and co-authors reported the positive values ΔH and ΔS [19].

#### 4.3.2. pH Effect

Antibiotics and HS are both ionic organic compounds containing one or more functional groups. Therefore, ionic species of these chemicals vary depending on pH values, which results in different sorption capacities of HS for antibiotics. Electrostatic interactions are often supposed to be the main interaction mechanisms between antibiotics and HS [14].

For macrolide TYL, a decrease in binding by HS with an increase in pH was demonstrated. The finding was related to the ionic species of antibiotic at different pH values [19]. At pH values below the pKa of antibiotics (7.1), the positively charged ions would be the major ionic species. Therefore, at acidic conditions, cation exchange was proposed as the dominant sorption interaction. At higher pH values, when species of the antibiotics would be the neutral molecules, electrostatic interactions between antibiotics and HS would weaken, and sorption would be dominated by hydrophobic interactions.

More often, a complicated pH-dependence of the binding of antibiotics by HS is reported [14,23,24,27,28,101]. This was demonstrated for fluoroquinolones (CIP [14], OFL [101], and NOR [28,101]), tetracyclines (OTC [27]), and sulfonamides (SDZ [27], SAA [24], SPY [24], and STZ [23]). The complicated pattern of the pH dependence of the HS–antibiotic interaction is mainly due to the antibiotics containing ionizable functional groups (carboxyl, hydroxyl, amino, or others). They provide the occurrence of antibiotics in the neutral, protonated, deprotonated, or partially protonated state (Figure 2, Table 2), whereas for HS a unidirectional increase in the deprotonation of functional groups with increasing pH is observed. Additionally, aromatic structures in HS might also be of importance [14,28]. As a result, depending on the antibiotic and HS, there may be opposite trends for the antibiotic–HS interaction in different pH ranges.

For fluoroquinolones in the range from acidic pH values to circumneutral (6–8), an increase in binding constants is usually observed followed by a decrease. The reported values of binding constants between CIP and soil or peat HA followed an ascending trend as pH increased from 4 to 6 (corresponds to pKa2 of CIP), whereas at higher pH there was either a decrease or no significant change in binding [14]. The observed tendency was due to the increasing deprotonation of the HS carboxylic acid groups with increasing pH values. For aquatic HS, however, a decrease or minimal change in the binding values as a function of pH in the range 4–8 was observed though the higher amount of the acidic functional groups detected in aquatic HS as compared with terrestrial ones. Thus, aromatic structures were hypothesized to favor the adsorption of CIP and the aromaticity of HS played a role in stabilizing CIP–HS complexes. Similar findings were reported by reported by Zhang and coauthors for the interaction of another fluoroquinolone, NOR, with coal-derived HA [28]. Over the pH range of 2.0–8.0, the sorption reached a maximum at pH 6.0 (corresponds to pKa2 of NOR), implying that the primary sorption mechanism was cation-exchange interaction between NOR^+^/NOR^0^ species and the negatively charged functional groups of HA. FTIR, and ^13^C NMR spectroscopy of NOR–HA complexes demonstrated that the piperazinyl moiety of NOR was responsible for sorption onto HA, while the carbonyl group and the aromatic structure of HA participated in complexing NOR.

The effect of pH on sulfonamide interaction with HS depends greatly on the antibiotic-specific speciation and cannot be generalized for all of them [24]. For example, from pH 4.5 to 6.0, the extent of the sorption of SAA, SPY, and SDM on Fluka HA declined, and the K_F_ values were lower by an average factor of 2. At pH 7.5, the K_F_ values of SAA and SPY were similar to those at pH 4.5, whereas the sorption of SDM to HA further declined, and K_F_ values were the smallest at pH 7.5 [24]. These findings were attributed to the different distributions of the studied antibiotics’ species (a cation, a neutral species, a zwitterion, and an acid anion) depending on the pH of the matrix. The calculated speciation showed that at pH 6.0 all three sulfonamides were basically neutral (94–99%), while at pH 4.5 considerable portions of SAA, SDM, and especially SPY were cationic (22%, 29%, and 62% respectively). At pH 7.5, the formation of anionic species was dominant for SDM (62%), but it was very low for SAA (<1%) and SPY (11%). The authors concluded that the sorption of cationic species was superior to the sorption of neutral and anionic species. The sulfonamide in the cationic form was proposed to interact most probably with HA through ion exchange. For neutral species of antibiotics, hydrophobic partitioning and physical sorption, such as van der Waals forces and hydrogen bridging, were suggested, while for acid anions, multivalent cation bridging was hypothesized. To confirm the supposed sorption mechanisms, differences in sorption nonlinearity were analyzed. They indicated slightly larger sorption specificity (i.e., n is very different from 1) of the ionic and especially of the anionic species and lesser specificity (i.e., n is close to 1) of the neutral sulfonamide molecules. Hydrogen bonding was assumed to be the main binding mechanism of sulfonamides to HS [24]. A decrease in the binding of SMZ and SDM to HS with an increase in pH was also demonstrated by Guo and coauthors [19,20].

For tetracycline OTC, K_b_ values were shown to be stable when the pH value increased from 4 to 6. Then, the binding constants rose with the increase of pH in the pH range 6–8. A further increase of the pH to 10 related to lower binding constants [27]. Tetracyclines are known to be hydrophilic (Table 2) and to carry a positive charge throughout the environmentally relevant pH range [110]. The cationic form is present up to pH ca. 5.5 and the zwitterion is present up to pH ≈9.5, dominating at pH 4–7. The net negatively charged ions are present at pH > 7. It should be noted, however, that at pH 8 the dominating ion of tetracyclines brings two negative and one positive charge, which are spatially separated and may act independently [110], while ions possessing only negative charges occur only at pH values above 8. As cation exchange is thermodynamically more favorable than physical partitioning-type processes, it may dominate even when only a small fraction of the aqueous-phase species exists as a cation [111]. Additionally, zwitterion and species possessing both negative and positive charges can be involved in the cation-exchange interaction between OTC and HS. The positively charged quaternary ammonium functional group of tetracycline TET may interact with the negatively charged sites of HS via cation exchange [104]. At an acidic pH condition 4–6, HS may exhibit an aggregated and bound state, forming a hydrophobic region because of charge neutralization [27], which hampers the interaction with hydrophilic OTC. When the pH increases from 6 to 8, more hydrophilic groups of HS are exposed, and the interaction is obviously aroused. A further increase of pH brings about the increase of electrostatic repulsion between OTC and HS due to the deprotonation of HS, and OTC is in the anionic form at these conditions [27].

#### 4.3.3. Effect of Ionic Strength and Multivalent Metals

Another way to further investigate the mechanism of antibiotic–HS interaction is to monitor the binding at varying ionic strengths. An increase in ionic strength is believed to cause shrinkage and a decrease in HS pore size, leading to a decrease in the total number of sorption sites. In addition, the shielding of the inert electrolyte in binding reactions containing opposite charged reactants emerged, which inhibited the formation of antibiotic–HS complexes [101]. Therefore, the electrostatic interactions between adsorbents and adsorbates decreased, whereas the hydrophobic interactions increased and the complexation did not have any obvious changes [112].

Negative effects of ionic strength on adsorption of antibiotics on HS are usually reported [20,22,101]. This phenomenon has been observed for fluoroquinolone CIP, ENR, NOR, OFL [101], macrolide TYL [20], and sulfonamide SMZ [20]. A considerable reduction in the binding values hints that interactions via H-bond formation and π–π and hydrophobic interaction might be important factors in the sorption of antibiotics on HS [20].

The effect of the addition of multivalent metal, in its turn, is of interest when the cation-bridging mechanism of antibiotic–HS interaction is considered. For tetracycline antibiotics, greater sorption in the Ca systems was demonstrated [18,104], especially under alkaline conditions [105]. A ternary complex formation tetracycline–metal–HS was proposed, while a negatively charged tricarbonylmethane keto-enol moiety of tetracyclines may interact with negatively charged sites of HS via cation bridging [104]. In addition, at high TET concentrations, the observed increase in the presence of calcium was demonstrated was due to the electrostatic interaction of positively charged tetracycline–Ca complexes with HS rather than due to the formation of ternary complexes [105]. Another study also reported an increase in the sorption of OTC on HS preloaded with Al and Fe(III) [113].

For fluoroquinolones, however, the effect of Ca^2+^ on antibiotic binding to HS is negative due to Ca^2+^ competing with the antibiotic for binding to the HS sites [28,101]. A similar effect has also been shown for macrolide antibiotics [105].

### 4.4. Discussion of the Putative Mechanisms of Antibiotic–HS Interaction: Main Issues

**Figure 3:**
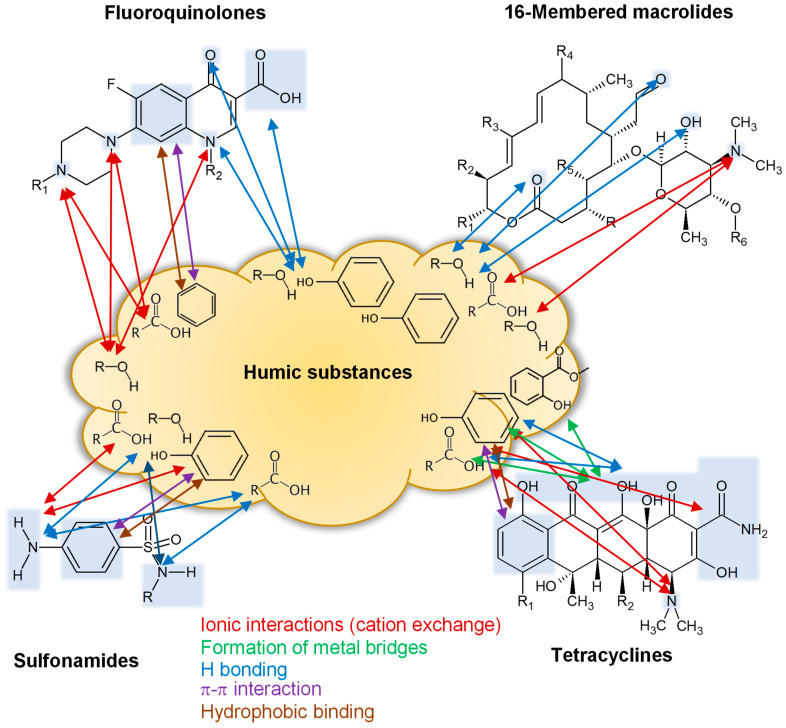
The main proposed mechanisms of antibiotics interaction with HS.

The analysis of published studies on the interaction of antibiotics with HS showed that the main mechanisms of interaction of antibiotics with humic substances are as follows (Figure 3):

- ionic interaction (cation exchange);
- formation of metal bridges;
- hydrogen bonding;
- Pi-stacking;
- hydrophobic interaction.

Some of the mechanisms listed above both have been repeatedly demonstrated for different types of antibiotics and proved using physical–chemical methods by studying the antibiotic–HS complexes.

However, a number of proposed mechanisms have not been sufficiently studied. An example of this is the possibility of interaction of HS with antibiotics by the mechanism of covalent binding. There are scarce works on this topic, although this type of interaction can lead to the complete detoxification of antibiotics in the environment. Gulkowska and co-authors demonstrated that sulfonamide antibiotic SMZ forms stable covalent bonds with quinone moieties in organic matter via nucleophilic addition reactions [38]. This process is initiated or occurs more intensively in the presence of oxidative enzymes such as peroxidase and laccase [34,108,114,115] and results in the formation of bound residues [108].

Another insufficiently studied field is the assessment of the dependence of the dominant interaction mechanism on the properties of HS. In most cases, commercially available HS of coal origin are studied. However, as ref. [14] very clearly showed, natural HS can show a very different behavior model, which is due to differences in their properties.

## 5. Humic-Based Sorbents for Antibiotics

The high binding ability of HS in relation to antibiotics has been demonstrated, which has made it possible to create a number of promising sorbents based on humic materials. To date, about 10 such sorbents have been described in the literature (Table 6).

In most cases, the developed sorbents are mineral or organic bases enriched with HS. HS coating introduces more oxygen-containing functional groups on the surface of the adsorbents, promoting their adsorption capacity. The developed humic-based sorbents were demonstrated to be efficient for the removal of fluoroquinolones and tetracyclines from environmental water systems [29] or polluted wastewaters [62]. Adsorption capacity usually reaches milligrams–tens of milligrams per gram of sorbent for fluoroquinolones and tens of milligrams for tetracyclines. The greatest value of adsorption capacity was reported for the calcium alginate/activated carbon/humic acid tri-system porous fibers, where it reached 266.78 mg of TET per g [63].

## 6. Environmental Consequences of Antibiotic–HS Interaction: Effect on ARGs Dissemination

One of the main problems arising from environmental pollution with antibiotics is the dissemination of ARGs [4,52]. In most cases, the relative abundance of antibiotic-resistance genes in relation to SU (*sulI*, *sulII*, *sulIII*), TE (*tetG*, *tetT*, *tetQ*, *tetX*, *tetW*), and MA (*ermB*) is analyzed.

In theory, the binding of antibiotics with HS should lead to a decrease in their bioavailability and, as a consequence, a decrease in the abundance of ARGs [49,50,51]. Chen and co-authors demonstrated diminished TET bioavailability as indicated by reduced expression of ARGs for a Gram-negative bacterium *Escherichia coli* in the presence of soil and peat HS at the concentration 5–50 mg OC L^−1^ [15]. Similar results were reported for another Gram-negative bacterium *Enterobacter aeruginosa* when a reduced antimicrobial activity of CIP–metal complexes by at least 2-fold due to Sigma-Aldrich HA presence in the broth media at 10 mg L^−1^ was detected [48]. However, no effect of HA on the toxicity of CIP–metal complexes was observed for a Gram-positive *Bacillus subtilis*, indicating that the HS adsorption on the bacterial outer membrane may also be of importance in hindering the antibiotic diffusion [48]. This observation is in line with the phenomenon that HS-degrading bacteria can be mostly affiliated with the Gram-negative *Proteobacteria* with an outer membrane containing lipopolysaccharides facilitating HS sorption [116]. The latter is of special importance as the potential host bacteria for ARGs was suggested to mainly belong to the Gram-negative phylum *Actinobacteria* [49].

**Figure 4:**
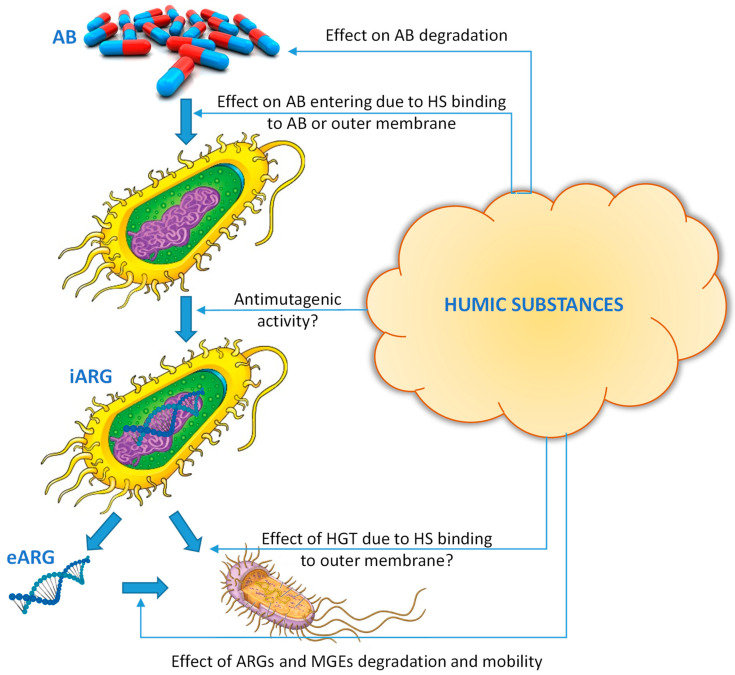
Putative ways of HS’ effect on ARG dissemination in the environment. AB—antibiotic; HS—humic substances; iARG—intracellular ARG; eARG—extracellular ARG; HGT—horizontal transfer; MGEs—mobile genetic elements.

The complexity of the ongoing interactions in the antibiotic–HS–bacteria systems leads to the ambiguity of the effect of HS on the dissemination of ARGs (Table 7, Figure 4).

Li and co-workers demonstrated a reduction of the most target ARGs in the Zn(II)-contaminated manure-soil samples by 11–88% due to treatment with struvite-HA-loaded biochar/bentonite composite (HMCC), while the abundance of *tetG* was enriched by 28% [49]. However, the system was too complex to precisely assess the role of HS and the authors explained the observed effects by reducing the amount of bio-available zinc due to Zn(II) sorption by HMCC, thus inhibiting the co-selection of ARGs. The immobilization of heavy metals was concluded to reduce the stress of metals on soil organisms, thus partially contributing to the control of soil ARGs. The reduction of extracellular AGR abundance was also found in the model experiments, where a significant enhancement of *tetA* gene photodegradation in the presence of Suwannee River Dissolved Organic Matter (SRDOM) was demonstrated due to photosensitization, resulting in a lowered transformation frequency [118].

Conversely, an increased abundance of ARGs in the presence of HS in the wastewaters or landfill leachates is usually reported [51,117], though the concentration of some fractions of HS, such as FA, is negatively correlated to the ARG concentration [51]. According to the authors’ assumptions, humic materials contribute heavily to the *sulI*, *sulII*, and *sulIII* gene abundance and dissemination because of their sorption of antibiotics and heavy metals [51]. Sun and co-authors also proposed that *tetA*, *tetW*, *sulI*, and *sulII* can adsorb with various organic colloidal particles [117]. On the other hand, the observed positive correlation between HS and ARG content may be due to the reported negative effect of humic materials on the decomposition of antibiotics in the environment [35,41,45,46]. The photolytic degradation of antibiotics can be impeded by HS due to the competition of photons and scavenging or quenching of reactive oxygen species [46]. However, the effect of HS on the photodegradation of antibiotics greatly depends on the properties of the humic material and an accelerating effect on the rate of antibiotics decomposition caused by HS is demonstrated more often [43]. Therefore, the characterization of HS at the molecular level can provide further insights into the assessment of photolysis for antibiotic elimination in natural waters in the presence of HS [46].

The complex influence of HS on the expression of ARGs is well illustrated by the results of ref. [50], where various effects were observed depending on the concentrations of HS and the target gene in the activated sludge during 4-chlorophenol wastewater treatment. The addition of 5 mg L^−1^ of HA induced 1.9- and 2.9-fold higher expression levels of *tetC* and *tetM*, respectively. Further increasing the concentration of HA to 25 mg L^−1^ did not influence the expression level of *tetM*, while the expression level of *tetC* decreased by 4.9-fold. The expression levels of *tetG*, *tetO*, *tetW*, and *tetX* were not influenced by the addition of 5 mg L^−1^ HA. However, except for *tetO*, increasing the HA concentration to 25 mg L^−1^ significantly reduced the expression levels of *tetG*, *tetW*, and *tetX*.

Another important issue is the possible interaction of HS with mobile-genetic elements (MGEs) associated with ARGs, including plasmids, integrons, transposons, and insertions. They can spread ARGs between microorganisms through horizontal gene transfer (HGT), which takes place through conjugation, transduction, and transformation [118]. The photosensitization of SRDOM was revealed to induce hydroxyl radical OH^•^ formation, thus enhancing plasmid strand breaks [118]. Wu and co-authors demonstrated that plasmid PBR322-mediated transformation frequency in *E. coli* could be inhibited by Sigma-Aldrich HA at 10–120 mg L^−1^ [119]. The study of the composition of landfill leachates revealed a positive correlation between the genetic markers of integrons *IntI1* and transposons *traA*, *tnp-A*/*Tn21* with HA concentration, but negative correlations with FA concentration. On the other hand, HA negatively correlated to the genetic marker of the insertion sequence IS-CR3, while FA correlated positively [51].

Overall, based on data available, one can conclude that there are still insufficient data to predict the effect of HS on the dissemination of ARGs. Despite a sufficient amount of evidence of intensive interaction of HS with antibiotics, this knowledge alone is not enough to understand the complex processes leading to a decrease or acceleration of the dissemination of ARGs. Obviously, in parallel with the assessment of binding, it is also necessary to take into account a number of other factors:

- toxicity of the antibiotic–HS complex for bacteria, which depends both on peculiarities of bacteria and HS;
- effects of HS on the rate of degradation of ARGs, which depends on HS properties;
- a final effect of HS on the efficiency of HGT.

Thus, in this area, it is necessary to conduct comprehensive studies of the interaction of HS with the potential host bacteria for ARGs, ARGs, and MGEs.

## 7. Prospects and Research Gaps

A review of the existing data showed that, to date, a significant number of studies have been conducted aimed at quantifying and establishing the mechanisms of the interaction of antibiotics with HS. The obtained data on the binding of antibiotics with HS provided the development of a number of effective sorbents for antibiotic purification of both natural waters and wastewater. A more intensive development of this direction may be hindered by the lack of a sufficient number of studies on the possibility of covalent binding of antibiotics to HS. In addition, when conducting research, high concentrations of both antibiotics and HS are often used, significantly exceeding those determined in natural objects. This makes it difficult to predict the behavior of antibiotics in natural environments. Therefore, an important direction in this area should be conducting research in close-to-natural conditions.

The lack of a unified approach for obtaining quantitative indicators of the interaction of antibiotics with HS greatly complicates the unification of data and, as a consequence, the meta-analysis, which would reveal the patterns of binding of antibiotics to HS. The situation is also complicated by the fact that research often does not pay enough attention to the characteristics of HS, although, as a number of studies have shown, the results obtained are directly determined by their properties. The study of the properties of HS is also a key point in assessing the environmental consequences of the formation of antibiotic–HS complexes such as the dissemination of antibiotic resistance, which is considered as a critical One Health issue being one of the biggest threats to health today [120]. A spread of ARGs on a local and global scale is a significant risk factor for global health. The presence and dissemination of microorganisms harboring acquired resistance determinants result in resistant superbugs in various environments. Therefore, the further study of the interaction of HS with antibiotics in terms of the development of technologies for wastewater treatment to lower the risk of the dissemination of ARGs is of great importance.

## Figures and Tables

**Table 1:** Reported concentration range for antibiotics of different classes in wastewaters (adapted from refs. [4,6]) and surface waters (adapted from ref. [66]). Values that exceed the predicted no-effect concentrations (PNEC) are shown in bold (PNEC values are based on eco-toxicology data generated by the member companies of Antimicrobial Resistance Industry Alliance and relevant peer-reviewed literature [81]).

Class	Wastewater, ng/L		Surface Water, ng/L	
	Min	Max	Min	Max
Africa				
FQ	40 (LEV, Kenya)	510 (CIP, Kenya)	40 (LEV, Kenya)	**14,331** (CIP, South Afica)
MA			1 (ERY, South Africa)	**1149** (ERY, Ghana)
SU			270 (SMZ, South Africa)	**53,828** (SMX, Mozambique)
TE			26 (OTC, Ghana)	68 (DOX, Ghana)
Asia				
FQ	240 (CIP, China)	**4960** (CIP, India)	3 (ENR, China)	6060 (NOR, Taiwan)
MA	7.5 (ROX, China)	**5542** (ERY, Vietnam)	1 (CLA, Japan)	**2910** (ERY, China)
SU	15 (SPY, Vietnam)	**9020** (SMX, India)	1 (SMX, Japan)	19,153 (SMR, Vietnam)
TE	682 (CTC, South Korea)	**32,000,000** (OTC, China)	84 (OTC, China)	**484,000** (OTC, China)
Australia				
FQ		**530** (CIP)	1150 (NOR)	**1300** (CIP)
MA			15 (ROX)	350 (ROX)
SU		**3750** (SMX)		
TE				
Europe				
FQ	185 (CIP, Spain)	**3700** (CIP, Italy)	90 (NOR, Spain)	**9660** (CIP, France)
MA	10 (ERY, Italy)	**780** (CLA, Italy)	10 (CLA, Spain)	**2330** (CLA, France)
SU	19 (SMX, Spain)	**1150** (SMX, Spain)	326 (SMX, Germany)	**11,920** (SMX, Spain)
TE			7 (OTC, Luxembourg)	1000 (TET, UK)
North America				
FQ		315 (CIP, USA)	30 (CIP, USA)	
MA	46 (ERY, USA)	145 (ERY, Canada)	66 (ROX, Canada)	180 (ERY, USA)
SU		**650** (SMX, USA)	60 (SMR, USA)	15,000 (SDM, USA)
TE			110 (MEC, USA)	1340 (OTC, USA)
South America				
FQ		156 (NOR, Brazil)	51 (NOR, Brazil)	119 (CIP, Brazil)
MA				
SU		9.9 (SMX, Brazil)	106 (SMX, Brazil)	218 (SMX, Bolivia)
TE			11 (TET, Brazil)	

Acronyms used for the antibiotics of different classes (PNEC in mg/L is indicated in parentheses): FQ: levofloxacin—LEV (1520); ciprofloxacin—CIP (450); enrofloxacin—ENR (1910); norfloxacin—NOR (120,000). MA: erythromycin—ERY (500); roxithromycin—ROX (6800); clarithromycin—CLA (250). SU: sulfamethazine—SMZ (no data); sulfamethoxazole—SMX (600); sulfapyridine—SPY (no data); sulfamerazine—SMR (no data); sulfadimethoxine—SDM (no data). TE: oxytetracycline—OTC (4700); doxycycline—DOX (25,100); chlortetracycline—CTC; meclocycline—MEC (no data); tetracycline—TET (3200).

**Table 2:** Some physicochemical properties of the antibiotics mentioned in this review.

Class	Antibiotic	Index	MM [6]	S, mg/L [6]	logK_OW_ [85]	pKa1	pKa2	pKa3	pKa4	pI
FQ	Ciprofloxacin	CIP	331.34	30,000	0.28	3.0 [86]	6.1 [86]	8.7 [86]	10.6 [86]	7.4 [87]
	Enoxacin	ENO	347.34	34,310	−0.2	6.0 [88]	8.5 [88]			7.4 [87]
	Enrofloxacin	ENR	359.4	53,900	0.27	3.9 [86]	6.2 [86]	7.6 [86]	9.9 [86]	6.9 [89]
	Fleroxacin	FLE	369.34	770	0.24	5.5 [6]	8.1 [6]			7.3 [90]
	Norfloxacin	NOR	319.33	178,000	0.46	3.1 [86]	6.1 [86]	8.6 [86]	10.6 [86]	7.4 [87]
	Ofloxacin	OFL	361.37	4000	−0.39	5.9 [6]	8.3 [6]			6.9 [87]
MA	Tylosin	TYL	916.1	5000	1.63	7.5 [86]				
SU	Sulfadiazine	SDZ	250.28	77	−0.09	2.1 [6]	6.5 [6]			4.7 [91]
	Sulfadimethoxine	SDM	310.33	343	1.63	2.1 [6]	6.3 [6]			
	Sulfamethazine	SMZ	278.33	1500	0.14	2.7 [6]	7.7 [6]			
	Sulfamethoxazole	SMX	253.28	610	0.89	1.9 [6]	10.6 [6]			3.3 [91]
	Sulfanilamide	SAA	172.2	7500	−0.62	1.9 [6]	10.6 [6]			
	Sulfapyridine	SPY	249.29	270	0.35	2.9 [6]	8.4 [6]			4.3 [91]
	Sulfathiazole	STZ	255.32	470	0.05	2.0 [86]	7.1 [86]			
TE	Oxytetracycline	OTC	460.4	313	−0.91	3.2 [86]	7.5 [86]	8.9 [86]		4–6 [92]
	Tetracycline	TET	444.43	1700	−1.37	3.3 [86]	7.8 [86]	9.6 [86]		4–6 [92]

Acronyms used for the antibiotics of different classes: FQ—fluoroquinolones, MA—macrolides, SU—sulfonamides, TE—tetracyclines. Acronyms used for the physicochemical properties: MM—molecular mass, S—solubility.

**Table 3:** Binding constants of HS to the selected antibiotics of different classes.

Class	AB	Reaction Media	Constants	Ref.
Stern–Volmer Constant K_SV_ / Binding Constant K_b_				
FQ	CIP	298 K, 303 K, 308K, 313K, 318 K; pH 7.0; 0.03 M phosphate buffer; Pahokee peat HA 0.25–2.5 mg L^−1^	nd/0.1719; 0.16; 0.1389; 0.133; 0.1151 L mg^−1^	[17]
		288 K, 298 K, 308 K, 318 K; pH 7.0; 0.001 M phosphate buffer; HA purchased from Alfa Aesar Chemical Company 0.2–2 mg L^−1^	0.149; 0.123; 0.118; 0.115 L mg^−1^/0.166; 0.125; 0.121; 0.121 L mg^−1^	[101]
		288 K; pH 3.1, 5.5, 7.2, 9.1; 0.001 M phosphate buffer; HA purchased from Alfa Aesar Chemical Company 0.2–2 mg L^−1^	0.062; 0.202; 0.123; 0.071 L mg^−1^/0.044; 0.202; 0.125; 0.059 L mg^−1^	[101]
		298 K; pH 7.0; 0.01/0.1 M phosphate buffer; HA purchased from Alfa Aesar Chemical Company 0.2–2 mg L^−1^	0.116; 0.081 L mg^−1^/0.118; 0.089 L mg^−1^	[101]
	ENO	298 K, 303 K, 308K, 313K, 318 K, pH 7.0, 0.03 M phosphate buffer, Pahokee peat HA 0.25–2.5 mg L^−1^	nd/0.0547; 0.0474; 0.042; 0.0353; 0.0262 L mg^−1^	[17]
	ENR	288 K, 298 K, 308 K, 318 K; pH 7.0; 0.001 M phosphate buffer; HA purchased from Alfa Aesar Chemical Company 0.2–2 mg L^−1^	0.16; 0.147; 0.135; 0.124 L mg^−1^/0.179; 0.154; 0.141; 0.124 L mg^−1^	[101]
		288 K; pH 3.1, 5.5, 7.2, 9.1, 11.0; 0.001 M phosphate buffer; HA purchased from Alfa Aesar Chemical Company 0.2–2 mg L^−1^	0.091; 0.27; 0.147; 0.05; 0.092 L mg^−1^/0.087; 0.268; 0.154; 0.053; 0.073 L mg^−1^	[101]
		298 K; pH 7.0; 0.01/0.1 M phosphate buffer; HA purchased from Alfa Aesar Chemical Company 0.2–2 mg L^−1^	0.117; 0.077 L mg^−1^/0.119; 0.069 L mg^−1^	[101]
	FLE	298 K, 303 K, 308K, 313K, 318 K, pH 7.0, 0.03 M phosphate buffer, Pahokee peat HA 0.25–2.5 mg L^−1^	nd/0.0642; 0.0544; 0.0531; 0.048; 0.043 L mg^−1^	[17]
	NOR	298 K, 303 K, 308K, 313K, 318 K, pH 7.0, 0.03 M phosphate buffer, Pahokee peat HA 0.25–2.5 mg L^−1^	nd/0.075; 0.0704; 0.0694; 0.0661; 0.0651 L mg^−1^	[17]
		288 K, 298 K, 308 K, 318 K; pH 7.0; 0.001 M phosphate buffer; HA purchased from Alfa Aesar Chemical Company 0.2–2 mg L^−1^	0.178; 0.163; 0.148; 0.138 L mg^−1^/0.194; 0.164; 0.147; 0.143 L mg^−1^	[101]
		288 K; pH 3.1, 5.5, 7.2, 9.1; 0.001 M phosphate buffer; HA purchased from Alfa Aesar Chemical Company 0.2–2 mg L^−1^	0.052; 0.136; 0.163; 0.118 L mg^−1^/0.044; 0.137; 0.164; 0.115 L mg^−1^	[101]
		298 K; pH 7.0; 0.01/0.1 M phosphate buffer; HA purchased from Alfa Aesar Chemical Company 0.2–2 mg L^−1^	0.163; 0.093 L mg^−1^/0.163; 0.097 L mg^−1^	[101]
	OFL	298 K, 303 K, 308K, 313K, 318 K, pH 7.0, 0.03 M phosphate buffer, Pahokee peat HA 0.25–2.5 mg L^−1^	nd/0.0332; 0.0339; 0.0289; 0.0297; 0.0304 L mg^−1^	[17]
		288 K, 298 K, 308 K, 318 K; pH 7.0; 0.001 M phosphate buffer; HA purchased from Alfa Aesar Chemical Company 0.2–2 mg L^−1^	0.127; 0.106; 0.098; 0.09 L mg^−1^/0.131; 0.109; 0.099; 0.097 L mg^−1^	[101]
		288 K; pH 3.1, 5.5, 7.2, 9.1, 11.0; 0.001 M phosphate buffer; HA purchased from Alfa Aesar Chemical Company 0.2–2 mg L^−1^	0.077; 0.051; 0.106; 0.022; 0.077 L mg^−1^/0.067; 0.07; 0.109; 0.015; 0.083 L mg^−1^	[101]
		298 K; pH 7.0; 0.01/0.1 M phosphate buffer; HA purchased from Alfa Aesar Chemical Company 0.2–2 mg L^−1^	0.091; 0.062 L mg^−1^/0.094; 0.06 L mg^−1^	[101]
SU	SDZ	288 K, 298K, 313 K; pH 7; water with NaOH/HCL to adjust pH; HA (no description) 2–20 mg L^−1^	13,174; 9811; 5055 L mol^−1^/12,784; 7183; 3223 L mol^−1^	[27]
		298K; pH 4, 8, 10; water with NaOH/HCL to adjust pH; HA (no description) 2–20 mg L^−1^	7282; 10,916; 10,363 L mol^−1^/6430; 9872; 8890 L mol^−1^	[27]
TE	OTC	288 K, 298K, 313 K; pH 7; water with NaOH/HCL to adjust pH; HA (no description) 2–20 mg L^−1^	7513; 5271; 3163 L mol^−1^/5533; 3123; 2051 L mol^−1^	[27]
		298K; pH 4, 8, 10; water with NaOH/HCL to adjust pH; HA (no description) 2–20 mg L^−1^	3894; 4043; 3946 L mol^−1^/3724; 4015; 3824 L mol^−1^	[27]
Distribution coefficient K_d_				
FQ	CIP	298 K; pH 6.0; 0.001/0.005/0.01/0.05/0.1 M CaCl_2_; HA purchased from Sinopharm Chemical Reagent 4000 mg L^−1^ (in solid)	445.31; 421.7; 400.34; 329.93; 277.17 L kg^−1^	[22]
	NOR	298 K; pH 6.0; 0.01 M CaCl_2_ + 0.01 M NaN_3_; compost HA 400 mg L^−1^ (in solid)	10.73 L g^−1^	[30]
	OFL	RT, pH 7.1, 0.01 M NaCl+200 mg L^–1^ NaN_3_; different fractions of HA from the Dianchi Lake sediment 5–42 mg L^−1^	70; 190; 60; 180; 50; 140; 40; 120 L kg^−1^	[21]
		298 K; 0.01 M CaCl_2_ +200 mg L^−1^ NaN_3_; HA (no description) 2–20 mg L^−1^ (in solid)	5570; 14,300 L kg^−1^	[26]
MA	TYL	278 K, 288K, 308 K; pH 3.0, 0.01 M KNO_3_ + 0.003 M NaN_3_; HA purchased from JuFeng Chemical Corporation (in solid)	174,6; 301.2; 620.7 L kg^−1^	[19]
		298 K, pH 3.0; 4.0; 5.0; 7.0, 0.01 M KNO_3_ + 0.003 M NaN_3_; HA purchased from JuFeng Chemical Corporation (in solid)	386.1; 352.7; 297.5; 268.4 L kg^−1^	[20]
		298 K, pH 3.0; 0/0.05/0.1 M KNO_3_ + 0.003 M NaN_3_; HA purchased from JuFeng Chemical Corporation (in solid)	457.6; 423.7; 375.4 L kg^−1^	[20]
SU	SMZ	288 K, 308 K, 318 K; pH 3.5; 0.01 M KNO_3_ + 0.003 M NaN_3_; HA purchased from JuFeng Chemical Corporation (in solid)	192.6; 243.2; 305.2 L kg^−1^	[19]
		298 K; pH 2.5, 3.5, 5.5, 7.5; 0.01 M KNO_3_ + 0.003 M NaN_3_; HA purchased from JuFeng Chemical Corporation (in solid)	235.6; 216.4; 189.7; 165.4 L kg^−1^	[20]
		298 K; pH 3.5; 0/0.05/0.1 M KNO_3_ + 0.003 M NaN_3_; HA purchased from JuFeng Chemical Corporation (in solid)	258.4; 176.5; 154.2 L kg^−1^	[20]
	SMX	298 K; pH 6.0; 0.001 M, 0.005 M, 0.01 M, 0.05 M, 0.1 M CaCl_2_; HA purchased from Sinopharm Chemical Reagent 4000 mg L^−1^ (in solid)	88.33; 84.64; 82.73; 62.98; 46.73 L kg^−1^	[22]
	STZ	292 K; pH 1.7, 2.5; 3.3; 4.9; 5.4; 6.0; 7.7; 10 mM ammonium phosphate at all pH except for 10 mM ammonium acetate at pH 4.9 and 5.4; coal HA 300, 800, 1800, 6400 mg L^−1^	Log(K_d_, L kg^−1^): 2.65; 2.36; 3.06; 2.66; 3.28; 2.81; 3.42; 2.88; 3.72; 3.12; 3.65; 2.98; 3.71; 2.84	[23]
TE	TET	RT; pH 5.0; 0.02M NaCl/0.02M NaCl + Zn 16.5 mg L^−1^; soil HA ca. 800 mg L^−1^ (in solid)	1300; 2700; 1600; 3100 L kg^−1^	[25]
		RT; pH 5.0; 0.02M NaCl/0.02M NaCl + Zn 16.5 mg L^−1^; coal HA ca. 800 mg L^−1^ (in solid)	1700; 3700; 5500; 9100 L kg^−1^	[25]
		RT, pH ~8, water with NaOH/HCL to adjust pH; leonardite HA 79.4 mg OC L^−1^	40,522 L kg^−1^	[16]
		RT, pH 7.0, LB media; Elliott soil HA, Pahokee peat HA, Waskish peat HA 9–91 mg L^−1^	12,036; 6732; 2750 L kg OC^−1^	[15]
Freundlich constant K_F_ / nonlinearity n				
FQ	CIP	RT; pH 4, 5, 6, 7, 8; 0.01 M acetate/phosphate buffer + synthetic freshwater; Elliott soil HA 10 mg L^−1^	91.59; 121.33; 166.72; 133.15; 172.38 mmol^1−n^ L^n^ kg^−1^/0.95; 0.99; 0.97; 0.98; 0.94	[14]
		RT; pH 4, 5, 6, 7, 8; 0.01 M acetate/phosphate buffer + synthetic freshwater; Pahokee peat HA 10 mg L^−1^	106.38; 159.6; 160.63; 149.99; 144.02 mmol^1−n^ L^n^ kg^−1^/0.96; 0.99; 0.97; 0.99; 0.99	[14]
		RT; pH 4, 5, 6, 7, 8; 0.01 M acetate/phosphate buffer + synthetic freshwater; Suwannee River HA 10 mg L^−1^	96.15; 84.47; 88.48; 83.34; 83 mmol^1−n^ L^n^ kg^−1^/1.02; 0.97; 0.98; 0.98; 0.97	[14]
		RT; pH 4, 5, 6, 7, 8; 0.01 M acetate/phosphate buffer + synthetic freshwater; Suwannee River FA 10 mg L^−1^	56.86; 49.06; 51.57; 34.27; 47.26 mmol^1−n^ L^n^ kg^−1^/1.13; 0.98; 0.93; 0.96; 0.96	[14]
		298 K, 308 K, 318 K; pH 6.0; 0.01 M NaCl+200 mg L^−1^ NaN_3_; HA purchased from Sinopharm Chemical Reagent 4000 mg L^−1^ (in solid)	1.48; 1.24; 1.01 mg^1−n^ L^n^ g^–1^/0.66; 0.65; 0.65	[22]
	NOR	298K; pH 2.0, 3.0, 4.0, 5.0, 6.0, 7.0, 8.0; 0.01 M CaCl_2_ + 0.01 M NaN_3_; coal HA 400 mg L^−1^ (in solid)	33.08; 37.14; 42.84; 57.9; 45.01; 51.09; 46.98 mmol^1−n^ L^n^ kg^−1^/0.44; 0.51; 0.46; 0.30; 0.41; 0.46; 0.40	[28]
		288K, 308K; pH 5.0; 0.01 M CaCl_2_ + 0.01 M NaN_3_; coal HA 400 mg L^−1^ (in solid)	64.81; 42.61 mmol^1−n^ L^n^ kg^−1^/0.41; 0.42	[28]
		298K; pH 5.0; 0.05 M CaCl_2_/0.1 M CaCl_2_ + 0.01 M NaN_3_; coal HA 400 mg L^−1^ (in solid)	31.06; 29.41 mmol^1−n^ L^n^ kg^−1^/0.46; 0.44	[28]
		298 K; pH 6.0; 0.01 M CaCl_2_ + 0.01 M NaN_3_; compost HA 400 mg L^−1^ (in solid)	90.01 mmol^1−n^ L^n^ kg^−1^/0.29	[30]
	OFL	RT, pH 7.1, 0.01 M NaCl+200 mg L^−1^ NaN_3_; different fractions of HA from the Dianchi Lake sediment 5–42 mg L^−1^	0.7449; 0.7176; 0.6888; 0.5412 mg^1−n^ L^n^ g^−1^/0.6051; 0.6312; 0.5395; 0.5668	[21]
MA	TYL	278 K, 288K, 308 K; pH 3.0, 0.01 M KNO_3_ + 0.003 M NaN_3_; HA purchased from JuFeng Chemical Corporation (in solid)	0.998; 1.385; 1.876 mg^1−n^ L^n^ g^−1^/0.23; 0.36; 0.61	[19]
		298 K; pH 3.0; 4.0; 5.0; 7.0, 0.01 M KNO_3_ + 0.003 M NaN_3_; HA purchased from JuFeng Chemical Corporation (in solid)	1.61; 1.432; 1.187; 0.986 mg^1−n^ L^n^ g^−1^/0.55; 0.44; 0.37; 0.32	[20]
		298 K; pH 3.0; 0/0.05/0.1 M KNO_3_ + 0.003 M NaN_3_; HA purchased from JuFeng Chemical Corporation (in solid)	1.923; 1.752; 1.487 mg^1−n^ L^n^ g^−1^/0.67; 0.60; 0.49	[20]
SU	SDM	294 K; pH 4.5, 6.0; 7.5; 0.025 Na formateformic acid buffer/0.2 M phosphate buffer; coal HA 2000 mg L^−1^ (in solid)	211; 124; 37 mmol^1−n^ L^n^ kg^−1^/0.87; 1.01; 0.26	[24]
	SMZ	288 K, 308 K, 318 K; pH 3.5; 0.01 M KNO_3_ + 0.003 M NaN_3_; HA purchased from JuFeng Chemical Corporation (in solid)	0.769; 0.942; 1.015 mg^1−n^ L^n^ g^−1^/0.77; 0.88; 0.92	[19]
		298 K, pH 2.5, 3.5, 5.5, 7.5; 0.01 M KNO_3_ + 0.003 M NaN_3_; HA purchased from JuFeng Chemical Corporation (in solid)	1.013; 0.839; 0.764; 0.687 mg^1−n^ L^n^ g^−1^/0.89; 0.85; 0.77; 0.69	[20]
		298 K, pH 3.5; 0/0.05/0.1 M KNO_3_ + 0.003 M NaN_3_; HA purchased from JuFeng Chemical Corporation (in solid)	0.986; 0.765; 0.681 mg^1−n^ L^n^ g^−1^/0.89; 0.75; 0.67	[20]
		298 K, 308 K, 318 K; pH 6.0; 0.01 M CaCl_2_ + 200 mg L^−1^ NaN_3_; HA purchased from Sinopharm Chemical Reagent 4000 mg L^−1^ (in solid)	0.47; 0.65; 0.95 mg^1−n^ L^n^ kg^−1^/0.61; 0.65; 0.62	[22]
	SAA	294 K; pH 4.5, 6.0; 7.5; 0.025 Na formateformic acid buffer/0.2 M phosphate buffer; coal HA 2000 mg L^−1^ (in solid)	49; 30; 48 mmol^1−n^ L^n^ kg^−1^/0.66; 0.56; 0.50	[24]
	SPY	294 K; pH 4.5, 6.0; 7.5; 0.025 Na formateformic acid buffer/0.2 M phosphate buffer; coal HA 2000 mg L^−1^ (in solid)	84; 44; 66 mmol^1−n^ L^n^ kg^−1^/0.45; 0.50; 0.49	[24]
	STZ	292 K; pH 1.7, 2.5; 3.3; 4.9; 5.4; 6.0; 7.7; 10 mM ammonium phosphate at all pH except for 10 mM ammonium acetate at pH 4.9 and 5.4; coal HA 300, 800, 1800, 6400 mg L^−1^	log(K_F_,mol^1−n^L^n^kg OC^−1^) (1.58; 1.6; 1.57; 1.45; 1.53; 1.19; 0.53 / 0.85; 0.80; 0.77; 0.73; 0.70; 0.66; 0.56	[23]
TE	TET	RT, pH 4.3, 0.01 M NaCl/0.1 M NaCl; Elliott soil HA 24 mg OC L^−1^	4290; 2270 mol^1−n^ L^n^ kg OC^−1^/0.99; 0.98	[18]
		RT; pH 5.0; 0.02M NaCl/0.02M NaCl + Zn 16.5 mg L^−1^; soil HA ca. 800 mg L^−1^ (in solid)	380; 380; 900; 900 mmol^1−n^ L^n^ kg^−1^/0.73; 0.73; 0.83; 0.83	[25]
		RT; pH 5.0; 0.02M NaCl/0.02M NaCl + Zn 16.5 mg L^−1^; coal HA ca. 800 mg L^−1^ (in solid)	700; 700; 6000; 6000 mmol^1−n^ L^n^ kg^−1^/0.76; 0.76; 0.98; 0.98	[25]
Langmuir constant K_L_ / Maximum adsorption b				
FQ	CIP	298 K, 308 K, 318 K; pH 6.0; 0.01 M CaCl_2_ + 200 mg L^−1^ NaN_3_; HA HA purchased from Sinopharm Chemical Reagent 4000 mg L^−1^ (in solid)	0.09; 0.077; 0.059 L mg^−1^/15.72; 14.45; 13.64 mg g^−1^	[22]
	NOR	298K; pH 2.0, 3.0, 4.0, 5.0, 6.0, 7.0, 8.0; 0.01 M CaCl_2_ + 0.01 M NaN_3_; coal HA 400 mg L^−1^ (in solid)	0.027; 0.024; 0.03; 0.042; 0.036; 0.049; 0.042 L μmol^−1^/338.29; 529.53; 466.75; 462.13; 461.95; 374.84; 391.47 μmol g^−1^	[28]
		288K, 308K; pH 5.0; 0.01 M CaCl_2_ + 0.01 M NaN_3_; coal HA 400 mg L^−1^ (in solid)	0.048; 0.043 L μmol^−1^/488.88; 377.61 μmol g^−1^	[28]
		298K; pH 5.0; 0.05 M CaCl_2_/0.1 M CaCl_2_ + 0.01 M NaN_3_; coal HA 400 mg L^−1^ (in solid)	0.05; 0.039 L μmol^−1^/271.75; 246.46 μmol g^−1^	[28]
		298 K; pH 6.0; 0.01 M CaCl_2_ + 0.01 M NaN_3_; compost HA 400 mg L^−1^ (in solid)	0.128 L μmol^−1^ / 340 μmol g^−1^	[30]
SU	SMZ	298 K, 308 K, 318 K; pH 6.0; 0.01 M CaCl_2_ + 200 mg L^−1^ NaN_3_; HA HA purchased from Sinopharm Chemical Reagent 4000 mg L^−1^ (in solid)	0.039; 0.046; 0.061 L mg^−1^/7.54; 10.21; 11.74 mg g^−1^	[22]

nd—no data. RT—room temperature. HA—humic acids.

**Table 4:** The calculated range of the binding constants of antibiotics to HS measured by different approaches.

AB	Stern-Volmer Model						Linear Model			Langmuir Model					Freundlich Model				
	K_SV_, L mol^–1^			K_b_, L mol^−1^			K_d_, L kg^−1^			K_L_, L mol^−1^		b, mol kg^−1^			K_F_, mol^1−n^L^n^/kg OC		n		
	Min	Max	N	Min	Max	N	Min	Max	N	Min	Max	Min	Max	N	Min	Max	Min	Max	N
Fluoroquinolones																			
CIP	20,543	66,931	10	14,579	66,931	15	277	445	5	19,549	29,821	0.041	0.047	3	65	3333	0.65	1.13	23
ENO				9100	18,999	5													
ENR	17,970	97,038	11	19,048	96,319	11													
FLE				15,882	23,712	5													
NOR	16,605	56,841	10	14,051	61,950	15	10,730	10,730	1	24,000	128,000	0.246	0.530	13	58	173	0.29	0.51	13
OFL	7950	45,894	11	5421	47,339	21	40	14,300	11						929	1289	0.54	0.63	4
Macrolides																			
TYL							175	621	10						1883	3672	0.23	0.67	10
Sulfanilamides																			
SDZ	3163	7513	6	2051	5533	6													
SDM															83	474	0.26	1.01	3
SMZ							154	305	10						1300	1938	0.67	0.92	10
SMX							47	88	5	9878	15,450	0.030	0.046	3	1058	2139	0.61	0.65	3
SAA															67	110	0.50	0.65	3
SPY															99	189	0.415	0.50	3
STZ							229	5248	14						3	40	0.56	0.85	7
Tetracyclines																			
OTC	5055	13,174	6	3223	12,784	6													
TET							1300	40,522	12						695	10,309	0.73	0.99	10

N—the number of experiments or different media studied.

**Table 5:** The main proposed mechanisms of HS and NOM interaction with antibiotics of different classes.

Class	AB	AB Reactive Moiety	HS Reactive Moiety	Ref.
Cation Exchange/Ionic Interactions				
FQ	CIP	Amino group in diazine cycle	Carboxylic	[14]
		nd	nd	[22]
		Amine	Hydroxy	[59]
	NOR	Piperazinyl	Carboxylic	[28]
SU	SMZ	nd	Carboxylic	[19,20]
	SMX	nd	nd	[22]
	SAA, SDM, SPY	nd	nd	[24]
	STZ	Aniline group	Carboxylic and phenolic	[23]
TE	TET	nd	Carboxylic	[18]
		Quaternary ammonium functional group	Carboxylic	[104]
		nd	Carboxylic	[25]
		Tricarbonylamide, phenolic diketone, dimethylamine	Carboxylic, Phenolic	[105]
Cation (metal) Bridging				
TE	TET	nd	Carboxylic	[18]
		Tricarbonyl methane keto-enol moiety	Carboxylic	[104]
		nd	Salicylate- and phthalate-like	[25]
		Tricarbonylamide, phenolic diketone, dimethylamine	Carboxylic, Phenolic	[105]
H-Bonding				
FQ	CIP	O atoms in the carbonyl group	Aromatic carboxyl or hydroxyl groups	[22]
		Carboxyl	Hydroxy	[27]
		O-H, C-H, -COOH, N-H	nd	[101]
	ENR	O-H, C-H, -COOH	nd	[101]
	NOR	O-H, C-H, -COOH, N-H	nd	[101]
		Carboxylic	Carbonyl	[46]
	OFL	O-H, C-H, -COOH	nd	[101]
SU	SMX	Sulfonamide N, heterocycle ring N	Carbonyl *	[55]
		Amide	Hydroxy	[22]
	SMZ	nd	O-alkyl structures	[20]
		nd	nd	[106]
	STZ	Amine	Carbonyl	[23]
	SAA, SDM, SPY	nd	nd	[24]
TE	OTC	nd	nd	[11,27]
	TET	nd	Carboxylic	[18]
		Polar groups	Acidic groups	[104]
		Hydroxyl, ketone, amino	Carboxylic, Phenolic	[25]
		Hydroxyl, Carbonyl groups (as H-bond acceptors)	nd	[105]
		Dimethylamine	Phenolic	[39]
π–π Interaction				
FQ	CIP	Aromatic ring	Aromatic rings	[14]
		nd	nd	[101]
	ENR, NOR, OFL	nd	nd	[101]
SU	SDZ	Aromatic ring	Aromatic rings	[27]
	SMZ	nd	nd	[20]
		Aromatic ring	Aromatic rings	[107]
TE	TET	Aromatic ring	Aromatic rings	[105]
Dipole-dipole interaction				
SU	SDZ	Pyrimidinyl	Polar structures	[108]
	SAA, SDM, SPY	nd	nd	[24]
Hydrophobic Binding				
FQ	CIP	nd	nd	[22]
	NOR	nd	Aromatic structures	[28]
	OFL	nd	nd	[21]
SU	SMZ	nd	nd	[19,106,107]
	SMX	Oxazole ring	Benzene ring	[22]
	SAA, SDM, SPY	nd	nd	[24]
TE	OTC	nd	nd	[11]
Covalent Binding				
SU	SDZ	Aromatic amine	Quinones	[108]
	SMZ	Aromatic amine	Quinones	[38]

*—model compounds. nd—no data.

**Table 6:** Humic-based sorbents for antibiotics.

AB	Sorbent	Ref.
Fluoroquinolones		
CIP	Magnetic biochar coated with HA	[29]
	Humic acid/cellulose nanocomposite beads	[59]
ENO	Magnetic biochar coated with HA	[29]
LEV	HA treated zeolite	[64]
NOR	Magnetic biochar coated with HA	[29]
Tetracyclines		
OTC	Fe_3_O_4_–HA–La composite	[61]
TET	Fe (III)-functionalized carbonized HA	[62]
TET	Calcium alginate/activated carbon/HA tri-system porous fibers	[63]
Isolated from *Streptomyces venezuelae*		
Chloramphenicol	HA loaded with nZVI particles	[65]

**Table 7:** The main effects of HS and NOM of the dissemination of antibiotic resistance.

AB Class	NOM	Media	Target ARG	Effect	Ref.
MA	HMCC 100 g/kg	Zn(II) contaminated manure-soil	*ermB*	Decreased abundance by 88%	[49]
SU	HA from landfill leachate	Landfill leachate	*sulI*, *sulII*, *sulIII*	Positive correlation between HA and ARGs concentrations	[51]
	FA from landfill leachate		*sulI*, *sulII*, *sulIII*	Negative correlation between FA and ARGs concentrations	
	HMCC 100 g/kg	Zn(II) contaminated manure-soil	*sulI*, *sulII*	Decreased abundance by 30–38%	[49]
	DOC 21-59 mg/L	Wastewater	*sulI*, *sulII*	Sorption on DOC	[117]
TE	HMCC 100 g/kg	Zn(II) contaminated manure-soil	*tetG*	Increased abundance by 28%	[49]
			*tetT*, *tetQ*, *tetX*, *tetW*	Decreased abundance by 11–58%	
	DOC 21-59 mg/L	Wastewater	*tetA*, *tetW*	Sorption on DOC	[117]
	Model DOM 20 mg/L	Water	*tetA*	Increased photodegradation rate constant by 1.8-fold. Transformation efficiency decreased	[118]
	HA 25 mg/L	Wastewater sludge	*tetC*, *tetG*, *tetW*, *tetX*	Down-regulation	[50]
			*tetM*, *tetO*	No effect or up-regulation	

Model DOM—Suwannee River Dissolved Organic Matter (2R101N) purchased from International Humic Substances Society (IHSS). FA—fulvic acids. DOC—dissolved organic carbon. HMCC—struvite-HA loaded biochar/bentonite composite; HA was provided by Sinopharm Chemical Reagent Co., Ltd. (Shanghai, China). Genetic markers of integrons (*IntI1*, *IntI2*, *IntI3*), of insertion sequences (*IS-26*, *IS-CR3*), of plasmids (*traA*, *trb-C*), and of transposons (*merA*, *tnp-A/Tn21*).

## Data Availability

The data presented in this study are available on request from the corresponding author.
